# Beneficial effects of hydroxychloroquine on blood lipids and glycated haemoglobin: A randomised interventional study in patients with rheumatoid arthritis and systemic lupus erythematosus

**DOI:** 10.1371/journal.pone.0312546

**Published:** 2024-10-28

**Authors:** Bengt Wahlin, Antje Braune, Elias Jönsson, Solveig Wållberg-Jonsson, Christine Bengtsson

**Affiliations:** Department of Public Health and Clinical Medicine/Rheumatology, Umeå University, Umeå, Sweden; Aswan University, Faculty of medicine, EGYPT

## Abstract

**Introduction:**

Hydroxychloroquine (HCQ) exerts a large reduction of cardiovascular risk in patients with inflammatory diseases, but the mechanisms are not fully known. The aim of this study was to study potential mechanisms for this.

**Methods:**

This interventional study (EudraCT 2014-005418-45) in 30 patients (23 with rheumatoid arthritis, 7 with systemic lupus erythematosus) investigates the effects of HCQ on cardiovascular risk factors and arterial stiffness in patients with inflammatory disease. Blood lipids, blood pressure, blood glucose, glycated haemoglobin (HbA1c) and arterial stiffness was assessed at initiation, after four weeks of treatment and after eight weeks of treatment with 200 mg HCQ daily.

**Results:**

After four weeks of treatment with HCQ, total cholesterol had decreased from 5.4 mmol/L to 5.1 mmol/L (p<0.001), low-density lipoproteins from 3,0 mmol/L to 2.7 mmol/L (p<0.001) and apolipoprotein B from 0.96 g/L to 0.90 g/L (p<0.01). Those levels remained unchanged after eight weeks of treatment with HCQ. Levels of triglycerides, high-density lipoproteins and apolipoprotein A1 remained unchanged during the study. HbA1c decreased in most patients, especially in patients with high levels at start of HCQ, but increased HbA1c was seen in patients with low levels at start of treatment with HCQ. No significant effect was seen on blood pressure or any measure of arterial stiffness.

**Conclusion:**

This study does not identify the mechanisms of cardiovascular risk reduction from HCQ. Arterial stiffness is not affected by HCQ. The impact of HCQ on HbA1c and blood lipids is rapid, but of modest magnitude, and these effects do not fully explain the reduced risk of cardiovascular disease seen in observational studies. The mechanisms of cardiovascular risk reduction from HCQ are yet not completely known.

## Introduction

Patients with rheumatoid arthritis (RA) and systemic lupus erythematosus (SLE) have an increased morbidity and mortality from cardiovascular disease (CVD) [1–4]. Both inflammation and traditional cardiovascular risk factors like smoking, male sex, hypertension and dyslipidaemia are associated with an increased risk of CVD in RA and SLE [3, 5]. Several observational studies have reported a decreased risk of CVD in patients with RA and SLE treated with chloroquine or hydroxychloroquine (HCQ) [6, 7]. These two compounds are highly similar, but due to a lower toxicity, HCQ is preferred over chloroquine [8]. In SLE, use of HCQ reduces flares and organ damage, and thus is recommended in all patients [8, 9], whereas HCQ is not much used in RA, due to a low efficacy in treatment of that disease [10, 11].

In observational studies, the reduced risk of CVD in patients with RA or SLE treated with chloroquine or HCQ corresponds to a more favourable blood lipid profile, a lower incidence of diabetes mellitus in those patients, and a better glycaemic control [7, 12–15]. However, the number of interventional studies on patients with inflammatory diseases confirming this as a pharmacological effect is low. Two studies of patients with RA have shown a significant improvement of lipid profile after initiation of HCQ [16, 17], whereas the results regarding glucose metabolism and insulin resistance after initiation of HCQ in patients with RA have been conflicting [16–18]. The number of interventional studies in patients with SLE is even more scarce. Two trials have found favourable effects on blood lipids in patients with SLE after initiation of HCQ [19, 20], but to the best of our knowledge, no prospective studies of glucose metabolism have been performed in patients with SLE. Similar to the findings in patients with RA and SLE, HCQ exerts beneficial effects in subjects without inflammatory diseases [21, 22].

Impaired arterial function can be identified as an increased arterial stiffness. Increased arterial stiffness is found both in patients with cardiovascular risk factors and established cardiovascular disease, and increased arterial stiffness is also predictive of future cardiovascular disease [10]. Assessment of arterial stiffness can be performed by non-invasive measurement of pulse-wave velocity (PWV), and several devices have been constructed for this purpose [11]. Arterial stiffness is reduced by antihypertensive medications and statins, a reduction that takes place within weeks from initiation of treatment [12, 13]. In cohort studies of women with SLE, use of HCQ was associated with a lower PWV [14, 15], but to the best of our knowledge, no interventional studies of the effect of HCQ on arterial stiffness in patients with SLE or RA have been published.

This interventional study was undertaken to study the potential beneficial effect of HCQ on cardiovascular risk factors in RA and SLE. The primary endpoint was to evaluate the effect of HCQ treatment on the traditional cardiovascular risk factors blood lipids, glucose metabolism, and blood pressure, whereas secondary endpoint was effects of HCQ on PWV.

## Materials and methods

### Design, setting and participants

The study reported was a non-blinded, interventional study.

**Fig 1:**
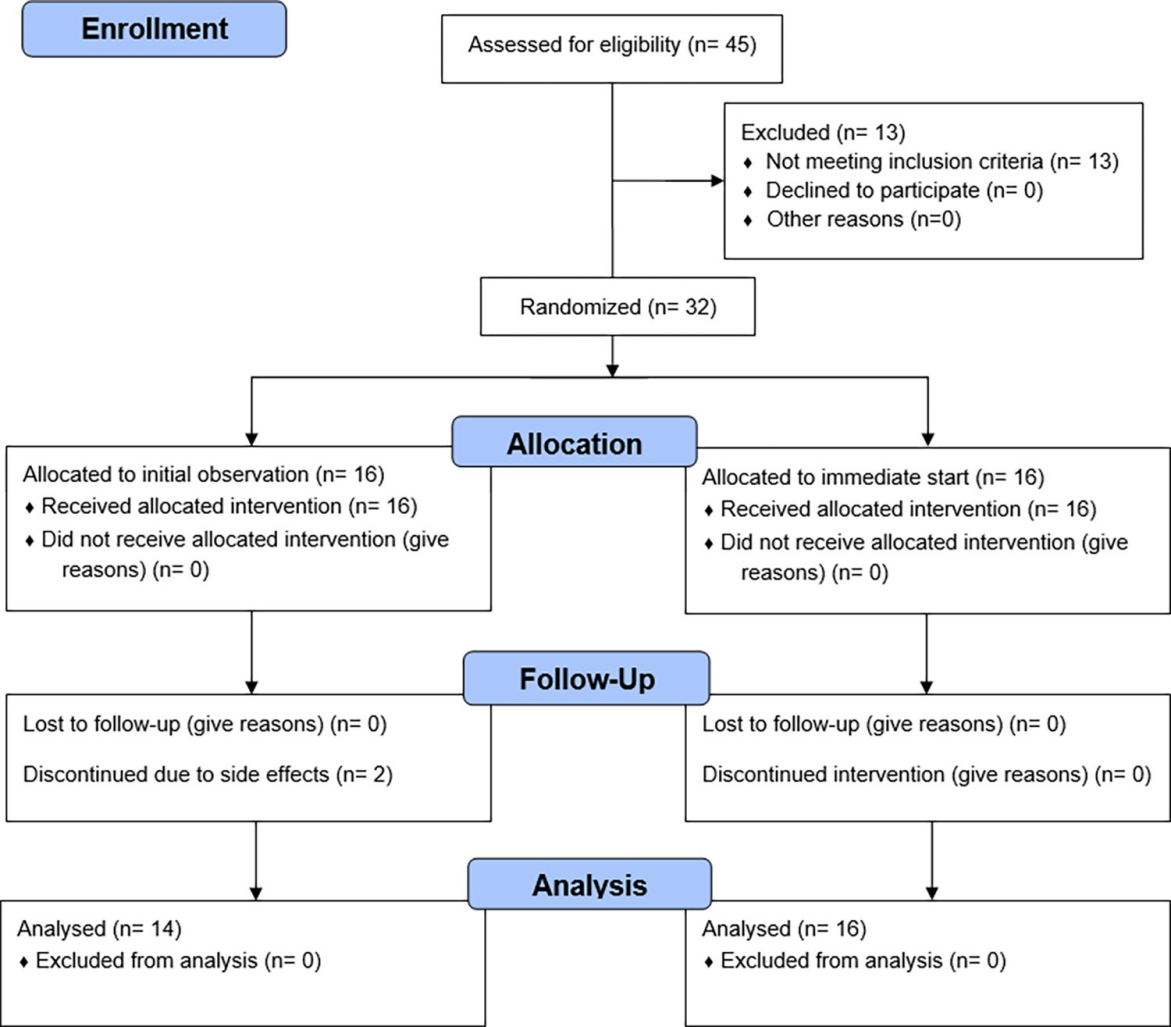
Enrollment of patients in the study.

In the four northernmost counties of Sweden, patients with SLE not treated with HCQ were identified by their clinical rheumatologists and invited to participate in the study. At the departments of rheumatology in Umeå and Östersund, patients with RA not treated with HCQ were consecutively invited to participate, if their treating rheumatologist regarded HCQ indicated. No other selection of patients was done. In all, 45 patients with RA or SLE were screened, of whom 25 patients with RA and 7 patients with SLE were eligible for the study, according to the inclusion and exclusion criteria. The period of inclusion started 25th August 2016 and ended 15th September 2016. Enrolment of patients is described in Fig 1. In short, the inclusion criteria stipulated the study participants to be male or female, 18–65 years of age, fulfilling the 2012 Systemic Lupus Erythematosus International Collaborating Clinics Group (SLICC) criteria for SLE [16] or 2010 ACR/EULAR criteria for RA [17], having prednisolone treatment less than 10 mg daily, and low to moderate disease activity (Disease activity score 28 joints (DAS28)) <4.6 in participants with RA, Systemic Lupus Erythematosus Disease Activity Index 2000 (SLEDAI-2k) <6 in participants with SLE [18, 19]). Exclusion criteria were chloroquine/HCQ treatment less than 250 days (five times the elimination half-life) before study inclusion visit, pleuritis or pericarditis, impaired visus and/or colour vision, auditory nerve damage, cardiomyopathy, atrioventricular block, blood pressure >160/95, diabetes, ongoing medical treatment with digoxin, short life expectancy due to morbidity, documented allergy or intolerance to HCQ, severe psychiatric condition, or other reason jeopardizing compliance with follow up. Patients were also excluded if medication with NSAID, prednisolone, antihypertensive medication or lipid-lowering treatment had been modified in the last two weeks, as were patients with adjustment of disease-modifying drugs in the last 6 weeks prior to screening. Pregnant patients were also excluded, and fertile female patients had a negative pregnancy test done before inclusion. At the screening appointment, SLEDAI-2k was assessed in patients with SLE and DAS28 in patients with RA, heart rate and blood pressure was measured, and present medication was registered. The participants attended their appointments at the Departments of Rheumatology in Östersund, Sundsvall, Sunderbyn and Norrlands Universitetssjukhus in Umeå.

Half of the participants included in the study were randomised to start treatment with HCQ after an initial observational period of four weeks, and thus had an additional measurement of all outcome variables four weeks before start of HCQ. This was done to study the potential effect of care. Randomisation was done using a computerised randomisation process, where half of the patients with RA and half of the patients with SLE in blocks of two within these groups were randomised to initial observation. The randomisation was not stratified for any other variable. The study was not blinded to neither participants nor assessors.

All participants provided their informed written consent, and the study was performed according to the Declaration of Helsinki and its amendments. The study was approved by the local board of ethics at Umeå University (2014-391-31M, 2015-206-32M) and the Swedish Medical Products Agency (EudraCT 2014-005418-45).

### Interventions

The participants in the study were treated with HCQ (Plaquenil®) 200 mg daily for eight weeks.

### Outcomes

At start of medication, after four weeks of treatment, and after 8 weeks of treatment, blood pressure and heart rate was measured, and after overnight fasting, blood samples were analysed for measurement of total cholesterol, high-density lipoproteins (HDL), low-density lipoproteins (LDL), triglycerides (TG), apolipoprotein A1 (ApoA1), apolipoprotein B (ApoB), lipoprotein (a), blood glucose, glycated haemoglobin (HbA1c) and CRP. All laboratory analyses were undertaken at the department of clinical chemistry at site, using routine methods. Furthermore, arterial stiffness and vascular function was assessed using Arteriograph® (TensioMed Ltd, Budapest, Hungary) [20], where aortic PWV, aortic and brachial augmentation index (Aix) and aortic pulse pressure (PP) was measured. The measurements were performed in accordance with the manufacturer´s instructions. In short, the participants were requested to refrain from tobacco and not drinking beverages containing caffeine or alcohol 12 hours before examination. The examination was performed in the dominant arm with the participant in supine position, in a silent room with air temperature 22–24°C.

The study protocol is available as S1 File, whereas the CONSORT check list is available as S1 Checklist.

### Sample size

The number of included participants was based on the assumptions of a 10 mmHg decrease in blood pressure, or 0.65 mmol/L decrease in total cholesterol from treatment with HCQ, numbers that would require 20 participants included in the study to achieve 80% power. The initial intention was to include 20 participants with SLE and 20 participants with RA.

### Statistical analyses

Change in variables over time was analysed by repeated measures ANOVA, Bonferroni corrected in pairwise comparisons. Associations between variables were analysed by Pearson correlation coefficient. Regarding the impact of care, changes in variables during the treatment period was compared between participants who initiated HCQ treatment immediately at start of the study, and those participants who had an initial four weeks of observation. Student´s t-test was done for this comparison. For variables where no significant difference in treatment effect was seen between the groups, the two groups were merged and analysed as one. P <0.05 was considered statistically significant in all analyses. All statistical analyses were performed using SPSS version 27 (Armonk, NY: IBM Corp).

## Results

Demographic data is presented in Table 1. Although every patient with SLE eligible for the study was included, the number of participants with SLE was only seven. Of the 32 participants included at screening, two did not complete the study and are not included in the results. Both were patients with an initial period of observation. No statistically significant difference between participants with an initial period of observation and those participants who started HCQ immediately was seen for any variable that changed statistically significant during the treatment. No serious or unexpected adverse events were observed during the study.

**Table 1:** Characteristics at start of treatment for the 30 RA-patients with RA and SLE who fulfilled the study. Numbers indicate mean (SD), except when indicated else.

**Demographic**		
	Age at inclusion, years	52 (11)
	Female, n (%)	26 (87)
**Cardiovascular risk factors**		
	Ever smoking, n (%)	7/27 (26)
	Current smoking, n (%)	1/27 (4)
	Systolic blood pressure, mm Hg	129 (19)
	Diastolic blood pressure, mm Hg	80 (14)
	Antihypertensive treatment, n (%)	6/27 (22)
	Statin treatment, n (%)	3 (11)
**Disease variables**		
	Disease duration, years	16 (9)
	Diagnosis RA, n (%)	23 (77)
	SLEDAI-2k, median (Q1-Q3)	4.0 (0–4.0)
	DAS28	2.7 (0.85)

DAS28: Disease activity score (28 joints). SLEDAI-2k: Systemic Lupus Erythematosus Disease Activity Index 2000

### Effects on lipids and glucose

**Fig 2:**
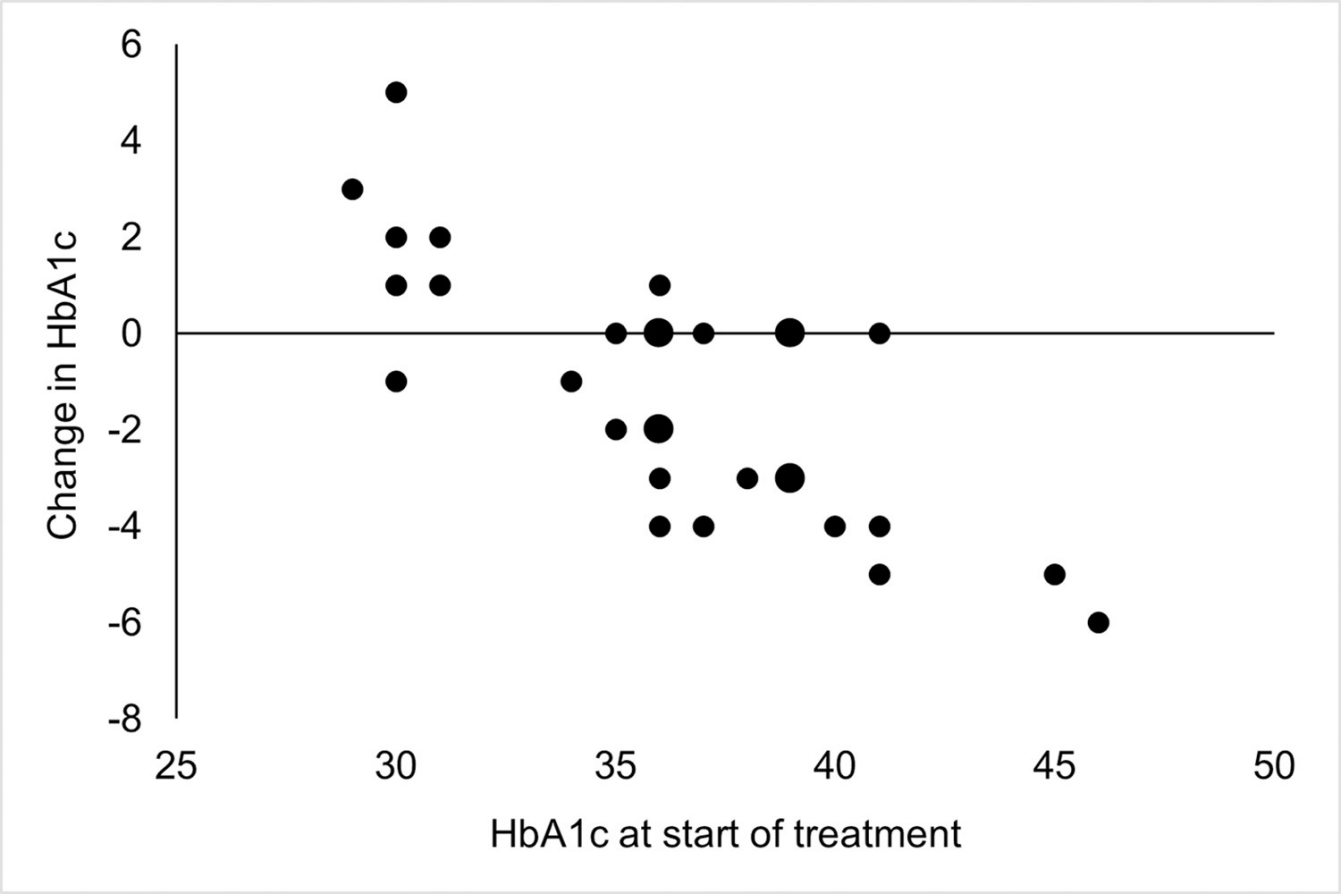
Change in HbA1c during treatment with hydroxychloroquine. Change in HbA1c during eight weeks of treatment with hydroxychloroquine is plotted against level of HbA1c at start of treatment in 30 patients with RA and SLE. Every small dot represents one patient, whereas large dots represent two patients with the same values of HbA1c.

A significant effect on blood lipids was seen four weeks after initiation of HCQ, with lower levels of total cholesterol and LDL (Table 2). In agreement with this, a significant effect was seen also in levels of ApoB and ratio ApoB/ApoA1. Levels of ApoA1, HDL and Lp(a) remained throughout the study unaffected by treatment with HCQ. Levels of HbA1c decreased significantly from treatment, although levels of glucose remained stable. Fig 2 illustrates change in HbA1c during treatment with HCQ, showing that HbA1c decreased in most patients, with a larger decrease in participants with higher levels of HbA1c. However, HbA1c increased in those participants with the lowest levels of HbA1c at start of treatment. The correlation between change in HbA1c and level of HbA1c at start of treatment with HCQ was highly significant (correlation coefficient -0.77, p<0.0001).

**Table 2:** Levels of lipid variables, metabolic variables and vascular variables at start of treatment with HCQ, after four weeks of treatment and after eight weeks of treatment. Values are presented as mean (SD).

Lipids	Start	4 weeks	8 weeks	p
Cholesterol, mmol/L	5.4 (0.83)	5.1 (0.78)	5.1 (0.83)	<0.001
LDL, mmol/L	3.0 (0.80)	2.7 (0.63)	2.7 (0.72)	<0.001
HDL, mmol/L	1.9 (0.51)	1.9 (0.52)	1.9 (0.54)	ns
Triglycerides, mmol/L	1.1 (0.56)	1.0 (0.42)	1.2 (0.66)	ns
ApoA1, g/L	1.7 (0.29)	1.7 (0.29)	1.7 (0.29)	ns
ApoB, g/L	0.96 (0.19)	0.90 (0.19	0.90 (0.17)	0.005
Ratio ApoB/ApoA1	0.58 (0.15)	0.57 (0.13)	0.55 (0.14)	0.04
Lipoprotein (a), nmol/L	54 (73)	51 (71)	0.51 (74)	ns
**Metabolic variables**				
HbA1c, mmol/mol	36.3 (4.3)	35.4 (4.0)	35.1 (2.9)	0.02
Glucose, mmol/L	5.2 (0.49)	5.2 (0.48)	5.2 (0.48)	ns
**Vascular variables**				
PWV, m/s	9.1 (2.6)	8.9 (1.9)	9.1 (2.3)	ns
Aortic systolic BP, mmHg	129 (24)	129 (29)	128 (33)	ns
Aortic pulse pressure, mmHg	49 (12)	51 (15)	50 (13)	ns
Brachial Aix, %	-9.1 (32)	-2.5 (51)	-7.4 (34)	ns
Aortic Aix, %	34 (17)	35 (17)	33 (17)	ns
Brachial systolic BP, mmHg	129 (20)	131 (23)	132 (21)	ns
Brachial diastolic BP, mmHg	80 (14)	80 (16)	81 (15)	ns
Heart rate, beats/minute	66 (10)	63 (10)	66 (9.1)	ns

Aix: Augmentation index. ApoA1: Apolipoprotein A1. ApoB: Apolipoprotein B. BP: Blood pressure. HbA1c: Glycated haemoglobin. HDL: High-density lipoprotein. LDL: Low-density lipoprotein. ns: not significant. PWV: Pulse-wave velocity. p for change during treatment, analysed with repeated measures ANOVA.

### Effects on vascular function

Measurements with Arteriograph® at start of HCQ, after four weeks of treatment and after eight weeks of treatment showed no significant changes in PWV, aortic or brachial Aix, aortic blood pressure or pulse pressure during treatment with HCQ (Table 2). Neither did systolic blood pressure, diastolic blood pressure or heart rate change during treatment with HCQ.

## Discussion

The main finding of this prospective, interventional study of HCQ in patients with RA and SLE, is a significant improvement of lipid status and decreased HbA1c after initiation of HCQ. This confirms results from observational studies and the few interventional studies that have been performed in patients with rheumatic diseases. The effect on blood lipids was seen already after four weeks of treatment and persisted throughout the eight-week period of follow-up, and it was of the same magnitude as have been seen in studies with longer duration of treatment with HCQ [7]. In agreement with the effects on levels of cholesterol, ratio of ApoB/ApoA1 decreased. The significant, but small, decrease in total cholesterol and LDL is of the same magnitude as in the data presented in the recent meta-analysis by Rempenault et al [7]. Although beneficial, these changes are modest and do not correspond to the reduced risk of cardiovascular disease seen in cohort studies, making it reasonable that other mechanisms contribute to the reduction in cardiovascular risk seen in observational studies of patients treated with HCQ.

Treatment with HCQ is an efficient glucose-lowering treatment in patients with type 2 diabetes, and HCQ is in India even approved for this purpose. Improvement in glucose metabolism could contribute to a lower cardiovascular risk also in non-diabetic patients treated with HCQ, since higher levels of HbA1c are associated with an increased cardiovascular risk not only in diabetic patients, but also in non-diabetic individuals [21]. Interestingly, HCQ had a dual effect on levels of HbA1c in our study of non-diabetic patients with RA and SLE. Not only decreased HbA1c in participants with higher levels at start of treatment with HCQ, with a larger decrease in the patients with higher levels at start, but HbA1c actually increased in the participants with lowest levels at start of treatment. This could be of interest in terms of cardiovascular risk in patients with diabetes, since diabetic patients with low levels of HbA1c suffer from an increased risk of cardiovascular disease and mortality [21]. This means that HCQ potentially could have dual favourable effects in patients with diabetes, if the normalising effect on levels of HbA1c is evident also in those patients. However, the relation between low levels of HbA1c and cardiovascular risk in non-diabetic patients has been inconsistent in previous studies, and numerically small when found [21, 22]. The results from our study do not tell to what extent effects on glucose metabolism explain the reduced risk of cardiovascular disease in patients treated with HCQ, but the changes in HbA1c are rather small. However, the duration of our study might be too short to evaluate the full effect of HCQ on HbA1c, since the average lifespan of erythrocytes is 120 days, and the duration of our study was only eight weeks.

Blood pressure is an important cardiovascular risk factor, that can be modified by pharmacological intervention. To the best of our knowledge, only one study of the effect of HCQ on blood pressure in patients with rheumatic diseases have been published. In that retrospective study of patients with RA, a lower blood pressure was seen after initiation of HCQ [23], in agreement with a prospective, placebo-controlled study of patients with metabolic syndrome [24]. A reduced blood pressure variability in patients treated with HCQ has been identified in other studies [25, 26]. In our prospective study, no effect on blood pressure was seen, and the design of the study did not permit analysis of the effects on blood pressure variability. The potential effect of HCQ on blood pressure needs to be studied further.

Furthermore, we measured arterial stiffness, as a possible mechanism of risk reduction beyond traditional cardiovascular risk factors. There are many processes leading to arterial stiffness. It is to some extent caused by changes in the arterial walls, where elastin is degraded and collagen is deposited, but also by endothelial dysfunction and inflammation [27]. The latter mechanisms probably explains why arterial stiffness responds quickly to pharmacological treatment; arterial stiffness is reduced within days after initiation of antihypertensive treatment [13]. However, we found no effect of HCQ on arterial stiffness in our study, and in light of the rapid effects on arterial stiffness from other medications, it is highly unlikely that our eight weeks long study was too short to detect an effect on arterial stiffness caused by endothelial dysfunction. Long-term effects on vascular stiffness from treatment with HCQ remains to be studied.

Overweight and obesity are associated with an increased risk of cardiovascular disease and altered blood lipids in the general population and patients with inflammatory diseases [28, 29]. We did not measure body weight or length in the present study, making body composition a possible confounder. However, the aim of the study was to analyse change after start of treatment with HCQ, and since HCQ does not affect weight, it is not likely to affect the results od the study. The effects were also seen already after four weeks of treatment and did not change further during the study.

In patients with RA and SLE, a high grade of inflammation is associated with an increased risk of cardiovascular disease [3, 5]. Studies in patients with RA have shown that the risk is diminished with pharmacological reduction of inflammation [30]. Although HCQ is a disease-modifying drug in treatment of RA, the effect of HCQ on inflammation in terms of ESR, CRP and swollen joints is modest, and not of the magnitude to make reduction of systemic inflammation *per se* a reasonable explanation to the impact of HCQ on cardiovascular disease in patients with RA [31]. In interventional studies of the effects of anti-inflammatory treatments on atherosclerotic cardiovascular disease, agents targeting interleukin 1 (IL-1), unlike agents with other modes of action, reduce the risk of atherosclerotic cardiovascular disease [32]. Interestingly, treatment with HCQ decreases IL-1 [33], making this a possible mechanism for the reduced cardiovascular risk. However, we did not assess IL-1 or other cytokines in our study. Thus, we cannot confirm that HCQ has a beneficial cardiovascular effect mediated through effects on IL-1 or other inflammatory mediators, but this remains to be studied.

This study has limitations. First, the number of participants is small, and both patients with RA and SLE are included, making the population studied heterogenous. The time of follow-up is short, and the outcome variables studied are risk factors, not cardiovascular events. The effects on blood lipids seen in the study are not clinically relevant, and do not explain the cardiovascular risk reduction from HCQ. On the other hand, it is an interventional study, compared to most previous studies that have been observational. The participants in our study are thoroughly examined and several measures of cardiovascular risk are assessed, and by this interventional study we can confirm some findings from previous observational studies.

## Conclusions

Hydroxychloroquine exerts rather small reductions in total cholesterol and LDL, and a beneficial effect on HbA1c. However, these effects are too small to explain the decrease in cardiovascular risk seen in patients treated with HCQ. As HCQ in our study exerts no beneficial effects on arterial stiffness or blood pressure, the reduced risk of cardiovascular disease in patients using HCQ is still not fully explained. Future studies of the cardiovascular effects of HCQ should examine IL-1.

## Supporting information

S1 Checklist CONSORT check list. (DOC)

S1 File Study protocol. (DOCX)
