# Supplementary material for: Beneficial effects of hydroxychloroquine on blood lipids and glycated haemoglobin: A randomised interventional study in patients with rheumatoid arthritis and systemic lupus erythematosus
Source: PLoS One. 2024 Oct 28;19(10):e0312546. doi: 10.1371/journal.pone.0312546 (PMC11515954; doi:10.1371/journal.pone.0312546)
Supplement: S1 File — (DOCX) [file pone.0312546.s002.docx]

***Improved cardiovascular risk factors and inflammatory markers in Rheumatoid Arthritis and Systemic Lupus Erythematosus?***

***New aspects of Hydroxychloroquine – an interventional study.***

**- a randomized controlled open trial**

**Study ID:** *HCQCVDRASLE*

**Version nr:** Version 1.4

**EudraCT number:** 2014-005418-45

**Sponsor name:**

Associate Professor Christine Bengtsson, MD, Ph.D, Dep of Public Health and Clinical Medicine, Umeå University, Rheumatology unit, Östersund Hospital, Sweden.

Professor Solveig Wållberg-Jonsson, MD, Ph. D., Dep of Public Health and Clinical Medicine, Rheumatology Clinic, Umeå University, Norrland University Hospital, Umeå, Sweden.

**Date of Protocol:** 15 Aug 2016

**Estimated study start:** 15 May 2016

**Estimated study end:** 31 Dec 2017

**AGREEMENT ON THE PROTOCOL**

The investigator agrees to conduct the study as outlined in this protocol with reference to national/local regulations and in accordance with the Declaration of Helsinki ^1^: Ethical Principles for Medical Research Involving Human Subjects.

The investigator agrees, by written consent to this protocol, to fully co-operate and allowing direct access to all documentation, including source data, by regulatory authorities.

Approved consent in writing:

| Signature: |  | Date: |  |
| --- | --- | --- | --- |
|  | Associate Prof Christine Bengtsson,  Dep of Public Health and Clinical Medicine, Umeå University,  Rheumatology Unit, Östersund Hospital, Sweden |  |  |

| Signature: |  | Date: |  |
| --- | --- | --- | --- |
|  | Prof Solveig Wållberg-Jonsson,  Dep of Public Health and Clinical Medicine, Umeå University, Rheumatology Clinic,  Norrland University Hospital, Umeå, Sweden |  |  |

TABLE OF CONTENTS

**1** **Abbreviation and Definition of Terms 5**

**2** **Attachments to Study Protocol 6**

**3** **Study team 6**

**4** **Synopsis 8**

**5** **Background and Rationale 13**

5.1 Background 13

5.1 Study Rationale 15

5.2 Study Timetable 15

**6** **Objectives of the study 15**

6.1 Primary Objective 15

6.2 Secondary Objective 15

**7** **Endpoints 15**

7.1 Primary Endpoint 15

7.2 Secondary endpoints 15

**8** **Study Design 16**

8.1 Description of Study Design 16

8.2 Justification of Study Design 16

8.3 Stop Criteria for the Study 17

**9** **Study Visits 17**

**10** **Study Population 19**

10.1 Number and selection of Subjects 19

10.2 Eligibility Criteria 19

10.2.1 Inclusion Criteria 19

10.2.2 Exclusion Criteria 19

10.2.3 Other Eligibility Criteria Considerations 20

**11** **Treatment within the study 20**

**12** **Withdrawal Criteria and Early Termination 20**

**13** **Study Assessments 21**

13.1 Demographic and Baseline Assessments 21

13.2 Assessments during the study 21

13.3 Biobank 21

**14** **Study medication and concomitant medication/therapy 21**

14.1 Study medication per treatment group 21

14.2 Administration follow-up of the medication: 21

14.3 Concomitant Medications 22

**15** **Adverse Event and Serious Adverse Event Collection, Recording and Reporting 22**

15.1 Definition of Adverse Events and Serious Adverse Events 22

15.1.1 Definition of an Adverse Event 22

15.1.2 Definition of a Serious Adverse Event 22

15.1.3 Definition of a Suspected Unexpected Serious Adverse Reaction 23

15.1.4 Disease-Related Events and/or Disease-Related Outcomes Not Qualifying as AEs/SAEs 23

15.1.5 Clinical Laboratory Abnormalities and Other Abnormal Assessments as AEs and SAEs 23

15.2 Reporting of Adverse Events 23

15.3 Time Period, and Frequency of Detecting AEs and SAEs/SUSARs 24

15.4 Pregnancies and Breast Feeding 25

**16** **Data Analysis and Statistical Considerations 25**

16.1 Sample Size Considerations 25

16.1.1 Data Sets and Populations 25

16.1.2 Interim Analysis 25

16.1.3 Key Elements of Analysis Plan 25

**17** **Study Administration 25**

17.1 Procedure for obtaining informed consent 25

17.2 Definition of Source Data 26

17.3 Quality Control 26

17.4 Quality Assurance 26

17.5 Study Closure 26

17.6 Records Retention 26

17.7 Case Report Forms 27

17.7.1 Rules for completing CRFs 27

17.7.2 Corrections to CRFs 27

17.7.3 CRF flow 27

**18** **Ethical and legal aspects 27**

18.1 Study ethics 27

18.2 Study approval, amendments and reporting to authorities 28

**19** **Data Management 28**

**20** **Finance and Insurance 28**

**21** **Reference 29**

Attachment 1. Schedule of Events

Attachment 2. Summary of Product Characteristics, Plaquenil

Attachment 3. SLEDAI-2k

Attachment 4. DAS 28

- **Abbreviation and Definition of Terms**

| **Abbreviation** | **Definition** |
| --- | --- |
| ACR | American College of Rheumatology |
| AE | Adverse Event |
| AR | Adverse Reaction |
| CRF | Case Record Form |
| DAS28 | Disease Activity Score 28 |
| HA | Hyaluronan |
| HCQ | Hydroxychloroquine |
| CVD | Cardiovascular diseases |
| HDL | High Density Lipoprotein |
| IUD | Intrauteral Device |
| LDL | Low Density Lipoprotein |
| MTX | Methotrexate |
| NA  ND | Not Applicable |
| ND | Not Done |
| NK | Not Known |
| PWV | Pulse Wave Velocity |
| NUS | Norrland University Hospital, Umeå, Sweden |
| RA | Rheumatoid Arthritis |
| SAE | Serious Adverse Event |
| SAR | Serious Adverse Reaction |
| SE | Side effects |
| SLE | Systemic Lupus Erythematosus |
| SLEDAI | SLE Disease Activity Index |
| SPC | Summary of Product Characteristics |
| SRF | Swedish Association of Rheumatology |
| SUSAR | Suspected Unexpected Serious Adverse Reaction |
| TC | Total Cholesterol |
| TG | Triglycerides |
| WOCBC | Women of Child Bearing Capacity |

- **Attachments to Study Protocol**

Attachment 1. Schedule of Events

Attachment 2. Summary of Product Characteristics of Plaquenil (Swedish)

Attachment 3. SLEDAI-2k

Attachment 4. DAS 28

- **Study team**

| **Name and title** | **Study function** |
| --- | --- |
| Christine Bengtsson, MD, PhD  Department of Public Health and Clinical Medicine, Umeå University,  Rheumatology Unit, Östersund Hospital,  831 83 Östersund, Sweden | Coordinating Investigator *(site 1);*  Sponsor |
| Elias Jönsson, MD,  Rheumatology Unit, Östersund Hospital,  831 83 Östersund, Sweden | Assistant investigator *(site 1)* |
| Karin Bengtsson  Rheumatology Unit, Östersund Hospital,  831 83 Östersund, Sweden | Research Coordinator *(site 1)* |
| Åsa Klockerud  Rheumatology Unit, Östersund Hospital,  831 83 Östersund, Sweden | Study Nurse *(site 1)* |
| Solveig Wållberg-Jonsson, MD, Ph. D, Prof Department of Public Health and Clinical Medicine, Umeå University, Rheumatology Clinic, Norrland University Hospital, 901 85 Umeå, Sweden | Principal Investigator *(site 2*);  Sponsor |
| Viktoria von Zweigbergk, Rheumatology Clinic, Norrland University Hospital, 901 85 Umeå, Sweden | Study Nurse *(site 2)* |
| John Svensson, MD  Department of Rheumatology, Sundsvall Hospital, 856 43 Sundsvall, Sweden, | Principal Investigator *(site 3)* |
| Therese Tiderman  Department of Rheumatology, Sundsvall Hospital, 856 43 Sundsvall, Sweden,  Sara Arnstigen  Department of Rheumatology, Sundsvall Hospital, 856 43 Sundsvall, Sweden,  Antje Braune, MD, PhD  Department of Rheumatology, Sunderby  Hospital, 971 80 Luleå, Sweden  Ulrika Åström  Department of Rheumatology, Sunderby  Hospital, 971 80 Luleå, Sweden | Study Nurse *(site 3)*  Study Nurse *(site3)*  Principal Investigator *(site 4)*  Study Nurse *(site 4)* |
| Marja-Liisa Lammi Tavelin, M.Sc. (Pharm.)  Clinical Trial Unit, Norrlands University hospital, 901 85 Umeå, Sweden | Study management, monitoring |

- **Synopsis**

| Study title | Improved cardiovascular risk factors and inflammatory markers in Rheumatoid Arthritis and Systemic Lupus Erythematosus? New aspects of Hydroxychloroquine – an interventional study (HCQCVDRASLE) |
| --- | --- |
| EudraCT number | 2014-005418-45 |
| Coordinating Investigator | Christine Bengtsson, MD, PhD Department of Public Health and Clinincal  Medicine, Umeå University, Rheumatology Unit, Östersund Hospital,  831 83 Östersund, Sweden |
| Study period | 01 May 2015 – 31 Jun 2016 |
| Development phase | Phase II |
| Objectives | Primary objective: To investigate the of HCQ treatment effects on traditional cardiovascular risk factor profile: blood lipid profile, B-glucose, blood pressure after 4 and 8 weeks in patients with RA and SLE.  Secondary objectives: To study the effect on vascular function, measured with pulse wave velocity (PWV) and inflammatory markers including CRP, cytokines, Calprotectin and Hyaluronan (HA).  To study the patient´s compliance to drug prescription and the occurring adverse events. |
| Study design | A randomized, controlled, open label, multicenter, interventional, phase II study. |
| Study population | Patients with RA (n=24) and SLE (n=24) from the Rheumatology departments in Umeå, Sundsvall, Sunderbyn and Östersund. |
| Duration of treatment | 8 weeks for both groups; for group 1 weeks 1 to 8, for group 2 weeks 5 to 12. After the study, the patients can continue to receive the study medication if their disease condition so requires (determined by the patient´s treating physician, prescription medicine). |
| Eligibility criteria | Inclusion criteria:   - Male or female, 18-65 years of age, who has given written consent for his/her participation to the study¸ Women of Childbearing Capacity (WOCBC) only if willing to comply with effective contraception methods during the course of the trial. Acceptable methods are such as oral contraceptives, contraceptive patches, contraceptive implant, vaginal contraceptive, double-barrier methods (for example, condom and spermicide), intrauterine device (IUD), hormonal IUD - ACR criteria for RA and SLE (4) - < 10mg Prednisolon daily doses - Low-medium disease activity = DAS28 <4.6-RA, SLEDAI-2k <6 SLE (Arthritis accepted)   Exclusion criteria:   - Antimalarial medication less than5 ½-t= *250 days* before study inclusion visit - High disease activity, where cortison adjustment is predictable - Pleuritis or Pericarditis – SLE - Impaired visus and/or colour vision - Auditory nerve damage - Cardiomyopathy - Brach block / atrioventricular block - Hypertension >160/95 - Diabetes - Ongoing medical treatment with digoxin - Re-introduction or recent (within 2 weeks) change in the dosing of NSAIDs, prednisolon, blood pressure lowering medication or antilipide treatment. For disease-modifying agents, within 6 weeks. - Short life expectancy due to other comorbidity - Documented allergy or intolerance to study drug - Severe psychiatric condition or other reason that jeopardize compliance with follow up. - On fertile females, pregnancy assessed by anamnesis and pregnancy test |
| Investigational Medicinal Product (IMP) | Hydroxychloroquine (ATC code P01BA02; Plaquenil ® film-coated tablet 200mg) |
| Dosage and route of administration | Plaquenil ® 200mg p.o. 1 tabl daily, 1+0+0+0 |
| Efficacy evaluation | For efficacy evaluation, subjects will be investigated for the following:   - Total Cholesterol (TC) - Triglycerides (TG) - Low density lipoprotein (LDL) - High density lipoprotein (HDL) - Apolipoproteins - Blood pressure - Pulse wave velocity - Blood-glucose- and HbA1c - Calprotectin in serum - Hyaluronan in serum - CRP - TNFr- 1 och 2, MCP-1, IL-2sR-alfa, IL-6, IL-17 samt IFN-gamma - Compliance to treatment   - Adverse Events  - Compliance to drug prescription |
| Safety evaluation  Safety evaluation | For safety evaluation, the subjects are monitored for compliance including adverse events occurrence. The compliance is measured by controlling drug accountability during patients´site visits. From the time a patient consents to participate in the trial until he/she has completed the trial (without any follow up period), all AEs will be reported in the CRF. All SAEs as defined in the protocol will be reported by filling out and sending the SAE form within 24 hours to *the monitor*. *The sponsor* will determine whether the event qualifies to immediate reporting to the regulatory authorities (SUSAR-reporting) or will be reported in the annual development safety update report (DSUR). SAEs occurring after trial termination must be reported if considered related to the treatment during the trial. |
| Statistical methods | Non-parametric tests will be used. Mann-Whitney´s test; when comparison between groups. Statistical testing of multiple assessments over time; Kruskal-Wallis or Friedmans test and Wilcoxons test. |
| Criteria for evaluation | All subjects enrolled will constitute the analysis population  Primary and secondary endpoints (except safety parameters) will be analyzed according to intention to treat.  Safety parameters will be analyzed as per protocol. |
| Interim analysis | No interim analysis is planned |

- **Background and Rationale**
- **Background**

The two inflammatory diseases Rheumatoid Arthritis (RA) and Systemic Lupus Erythemato-sus (SLE) have the major comorbidity problem of Cardiovascular diseases (CVD) in common. The risk for CVD is overall more than doubled in both RA (1) and SLE compared with population/controls. In SLE, this risk for CVD is emphasized and noted 8 times increased in middle aged women (2, 3). In our further studies of surrogate markers and risk factors for CVD, we could show both an increased Intima-Media thickness and decreased endothelial function in RA compared with controls (4, 5). In SLE we have shown an increased vascular stiffness compared with controls (6). Traditional risk factors (e.g. dyslipidemia and hypertension) for CVD are increased in RA and SLE although with diverging results (7-10).

Klorokin medication is registered as RA treatment and recently shown beneficial in combination with Sulfasalazin and MTX compared with TNF (11). In SLE Klorokin has dual effects in lowering disease activity (especially skin and joint involvement) and preventing flare (12, 13). Retrospective studies of SLE have demonstrated an association between use of Klorokin and improved lipid profile (14), vascular stiffness (15) as well as risk of thrombosis (16). An association between improved blood lipid profile and Klorokin treatment have also been noted in RA (17,18). One interventional noncontroled study showed improvement of cholesterol and low density lipoprotein (LDL) levels in SLE patients on Hydroxychloroquine (HCQ) medication (19). There is also a growing body of evidence that HCQ has a positive influence on glucose metabolism (Hage MP et al 2014).

Hyaluronan (HA), a major component of the extracellular matrix (ECM) has been known as lubricant and shock absorbing in joints. It has also been shown that high molecular size HA (400-2000 kDa) has several regulatory and structural functions (20-22) as well as being anti-inflammatory and immunosuppressive (23,24). Low molecular size HA (10-500 kDa) is in-volved in the induction of NFκB and increase of cyto-and chemokines (25). High molecular size HA can also activate the Toll-like receptors an initiate immune response (20, 26, 27).

The activity of NFκB is known to be decreased in SLE ( 28) leading to a lack of inhibition of apoptosis and promoting chronic inflammatory responses ( 29). The Toll-like receptors play a major role in the pathogenesis of SLE where Hydroxychloroquine (HCQ) has been shown to have an antagonistic effect (30). The surface molecule CD44 participates in T-cell adhesion and migration is increased in SLE patients (28). Among the rheumatic diseases, an epidermal imbalance between HA and its CD44 receptor in patients with Psoriatic Arthritis has recently been demonstrated (31). Both patients with Dermatomyositis and Lupus nephritis have in-creased serum levels of HA (32, 33). However the knowledge of LMW-HA in SLE is scarce, why we find it of great interest to study the serum levels of low disease activity-SLE further-more serum levels of LMW-HA in SLE patients before and after HCQ treatment.

We performed a pilot study as a student project 2013:

Objectives: In earlier, retrospective, studies chloroquine has been found associated with more favorable risk factors; reduced pulse wave velocity (PWV), inflammation, cholesterol and fasting glucose. The purpose of this pilot study was to longitudinally study these effects.

Methods and materials: Nine SLE-patients with low disease activity, median age 54 (44-64), no more than 10 mg prednisone daily and without antimalarials were recruited to our study. Eight patients completed one follow-up, 7 completed the whole study. Two of them had dia-betes treated with insulin. Analysis and measurements were performed before and after 4 and 8 weeks of treatment with 200 mg hydroxychloroquine (HCQ) daily dose. Laboratory tests of inflammation, lipids and glucose were analyzed according to routine. Calprotectin, a novel marker for disease activity, in serum was detected with ELISA in the department of clinical immunology in Umeå. PWV was measured oscillometrical, with the validated device Arterio-graph®.

Results: Beneficial effects on LDL- (3,4±0,9 vs. 3,0±1,0 mmol/l, p=0,016) and HDL-cholesterol (1,6±0,3 vs. 1,8±0,5 mmol/l, p=0,031) were seen after 4 weeks of treatment. After 8 weeks CRP was lowered (5,6±7,4 vs. 2,1±1,8 mg/l, p=0,094), while calprotectin initially increased but after 8 weeks was slightly lower than baseline (523,7±309,8 vs. 716,3±522,4 ng/ml, p=0,023 and 523,7±309,8 vs. 499,5±286,0 ng/ml, p=0,742 respectively). PWV was unchanged, but the youngest patient in the study did show a marked lowering of PWV. We could not show any correlation between PWV, CRP and calprotectin. Among patients without diabetes HbA1c was lowered (37,8±3,3 vs. 35,8±3,8 mmol/mol, p=0,063) while fasting glucose showed no change.

Conclusion: In this pilot study we were able to show, despite few patients, that HCQ affects LDL- and HDL-cholesterol in 4 weeks in SLE-patient, also with low prednisone dosage. Ben-eficial effects on inflammation and glucose levels were also noted. However, no effect on vascular stiffness was shown, which might be related to the relatively high age and substantial amount of comorbidity among the patients.

**Preliminary results from HA-pilot study**

Dispersity of hyaluronan was analysed with GEMMA in serum from 5 patients (A-E) and 5 age matched controls (K1-K5). The controls size profiles were almost identical and an average was calculated to be used for comparison. Blood samples were collected from patients the beginning of the pilot study (#1) and after 8 weeks of HCQ treatment (#3). Hyaluronan was extracted from serum and analysed with GEMMA. Three of the patients initially showed an increased concentration of a hyaluronan size displayed as a peak between 40 to 45 seconds. This size is also present on control samples as a distinct peak. Sample taken after 8 weeks of HCQ treatment were available from two of these patients (Fig. 1 a, b). They showed a decrease of this size of hyaluronan, even below that observed in the controls. One of these patients also showed a decrease of a size of hyaluronan, displayed as a peak about 30 seconds (Fig. 1b).

However, two of the patients show no significant change in the size spectra of hyaluronan, either in the beginning of the study, or after 8 weeks (Fig. 1c). This shows that if a patient does not have an increased amount of this size of hyaluronan in the blood, then HCQ is unable to lower the normal concentration of this size of hyaluronan.

Is HCQ affecting a process not active in all patients and can this be a way of determine which patients who benefits from HCQ treatment?

a b c

- **Study Rationale**

The hypothesis is that hydroxychloroquine (HCQ) has positive effects on risk factors of CVD in RA and SLE. We will also investigate serum levels of HA before and after HCQ treatment to confirm the hypothesis from the pilot study, that HCQ-medication normalize the concentration of HA.

- **Study Timetable**

The study is estimated to start during Q2 2015. Recruitment time is estimated to 2-3 weeks. The study period is 8 weeks for group 1 and 12 weeks for group 2, however the treatment period is 8 weeks for each group. The study period altogether is 12 weeks.

- **Objectives of the study**
- **Primary Objective**

The primary objective is to investigate the effects of HCQ treatment on traditional cardiovascular risk factor profile: blood lipid profile, B-glucose, blood pressure after 4 and 8 weeks of treatment in patients with RA and SLE.

- **Secondary Objective**

The secondary objective is to study the effect on vascular function, measured with pulse wave velocity (PWV) and inflammatory markers including, CRP, TNFr- 1 and 2, MCP-1, IL-2sR-alfa, IL-6, IL-17, IFN-gamma, Calprotectin, Hyaluronan (HA), compliance and AE.

- **Endpoints**
- **Primary Endpoint**

The primary endpoint is to evaluate the effect of treatment on the blood lipids; Total Cholesterol (TC), Triglycerides (TG), Low density lipoprotein (LDL), High density lipoprotein (HDL) and apolipoproteins in patients with RA and SLE.

- **Secondary endpoints**
- Pulse wave velocity analyses after HCQ-medication
- Blood pressure
- Blood-glucose- and HbA1c levels after HCQ-medication
- Calprotectin levels in serum after HCQ-medication
- Hyaluronan size – and concentration in serum after HCQ-medication
- CRP
- TNFr- 1 and 2, MCP-1, IL-2sR-alfa, IL-6, IL-17, IFN-gamma
- Compliance to treatment
- Adverse events
- Compliance to drug prescription
- **Study Design**
- **Description of Study Design**

This is a randomized, controlled, open label, multi center, interventional study. We will evaluate the effects of HCQ-medication on cardiovaskular risk factors and inflammatory markers in RA and SLE.

- **Justification of Study Design**

**Risk-benefit for the individuals**:

Hydroxychloroquine is approved for treatment of the diagnosis SLE and RA to prevent flare and to lower disease activity, which is the main reason for the prescription of the medication. Hydroxychloroquine has been approved for clinical use for decades and its safety profile is well-documented.

The most common side effects of hydroxychloroquine are nausea and diarrhea, which often improve with time or by taking the medication with food. Less common side effects include skin rashes, changes in skin pigment (such as darkening or dark spots) or hair changes (bleaching or thinning of hair), sensory-motoric disorder, anorexia, tinnitus, dizziness and weakness.

In rare cases, hydroxychloroquine has caused visual changes or loss of vision, but such vision problems are more likely to occur in individuals taking high doses for many years, in elderly patients, or in those with significant kidney disease.
Also neurological hearing disorders (after long-term treatment) and liver defects have been reported in rare cases.

With unknown reporting frequency adverse reactions such as anemia, hypoglycaemia, hearing impairment, psychosis and cardiomyopathy have been reported.

For complete safety information on hydroxychloroquine please see Attachement 2. Summary of Product Characteristics of Plaquenil (Swedish).

The patients in highest risk for developing severe adverse reactions are excluded from the study (see sections 10.2 “Eligibility Criteria” and 14.3 “Concomitant medication and non-drug therapies”). The adverse events and reactions occurring during the study are closely monitored (see sections 12. “Withdrawal Criteria and Early Termination” and 15. Adverse Event and Serious Adverse Event Collection, Recording and reporting”) and the actions will be taken to treat the reactions and mitigate the risk for worsening. The short period of drug treatment (8 weeks) will decrease the likeliness of developing long-term treatment adverse reactions.

To receive information in writing about the increased risk of CVD can worry the patient. However, we do have a strategy in answering the patients´ questions and also a structured plan for dealing with cardiovascular risk factors that need direct medical intervention. The patients will be able to reach the study nurse by phone at every site and if needed, the investigator is available for answers. If risk factors for CVD are in need of direct investigation or medical treatment, the patient will be remitted for this as in clinical praxis.

Taking part of the study will take a sufficient amount of time for the patient. However, the study is optional and contains a rigorous amount of tests regarding cardiovascular risk factors, which is of potential benefit for the patients.

Twenty patients in each group are needed for a 80%-ig power of lowering blood-pressure 10 mmHg. Lowering LDL or TC 0.65 mmol/L with SD 0.7 gives a power of 0.816 when 20 patients in each group are included.

- **Stop Criteria for the Study**

If any new alarming information regarding the safety profile of hydroxychloroquine in the studied population comes to the spnsoror´s knowledge during the trial, immediate actions will be taken to assess whether the study is to be stopped. See also section 17.5 “Study Closure”.

- **Study Visits**

Patients with RA and SLE will be recruited from the Rheumatology departments in Umeå, Sundsvall, Sunderbyn and Östersund. Our intention, if possible, is to recruit the same amount (n=24) of patients from each diagnoses. Patients full filling the classification criteria for RA (ICD-10 M05.9) and SLE (ICD-10 M32.9) and without ongoing HCQ-medication, will be invited to participate in the study. Information about the study, with informed consent and return envelope, will be sent by post, to the patients. The patient information will include name and phone number to nurse and physician from each center, for patients to ask any questions about the study. If the patient agrees to participate, a screening visit is booked with-in 2-3 weeks.

Two-three weeks before week 0 a **screening** visit is performed. After screening, randomization is performed.

Group 1 starts HCQ-medication at week 0. **Assessments** are performed at week 0, 4 and 8. After this the patients may continue the study medication outside the study due to the diagnose RA or SLE.

Group 2 starts HCQ-medication at week 4. **Assessments** are performed at week 0, 4, 8 and 12. After this the patients may continue the study medication outside the study due to the diagnos RA or SLE.

See attachment 1. Schedule of events for more detailed description of the study procedures.

Each visit for assessments with blood samples and measurements of PWV takes 30 minutes + 30 min.

The medical treatment period within the study is 8 weeks for both groups. But the medication may continue due to the disease and diagnose.

**Screening:** Informed consent, inclusion and exclusion criteria are controlled (10.2). Disease activity is investigated regarding the diagnosis, **SLEDAI-2k** (attachment 3) for SLE and **DAS-28** (attachment 4) for RA. Medical history and present medication is recorded. Vital signs are investigated. For pre-menopausal women a pregnancy test is performed. Blood samples for analyses regarding disease activity is included in each diagnose-specific index (SLEDIA-2k and DAS 28). Analyses regarding blood lipids will also be performed (see also schedule of events). The screening visit takes 1.5 hour. After screening visit the patients are coded and randomly assigned to group 1 or group 2.

**SLEDAI-2k** is a disease activity index in SLE calculated from count of activity in all organs involved in this disease: Neuropsychiatric signs, visual disturbance, lupus headache, cerebrovascular incidence, vasculitis, arthritis, myositis, skin manifestations, serositis, signs of nephritis, hematological or serological manifestations and fever are recorded in this index (34).

**DAS-28** is a disease activity index in RA, used in every day-clinic. It is calculated from investigation of 28 joints, according to “swollen joints” and “tender joints”. Knees, shoulders, elbows, wrists, metacarpo-phalangial and proximal interphalangial joints are included in DAS-28. Patients global health, sedimentation rate, C-reactive protein-level and Health Assessment Questionnaire are also included in the calculation of DAS-28 (35).

Blood samples are drawn after overnight fasting. Analyses of blood lipids, b-glc and HbA1c are performed at accredited Clinical Chemical laboratory at each site. Serum is sent to Clinical Immunology, NUS, as routine and transported to the Rheumatology department for storage in biobank (SoS443) freezer at -20◦C for analyses of, TNFr- 1 and 2, MCP-1, IL-2sR-alfa, (Wållberg-Jonsson et al 2008, unpublished data in manuscript, Södergren A et al), IL-6, IL-17, IFN-gamma (Lopez-Pedrera et al, 2010), Calprotectin (36) (by ELISA analysis)and HA after the study is closed. Pulse wave Velocity is measured with Arteriograf®.

Measuring PWV (37) with Arteriograf® is a noninvasive technique, were the pulswaves are registered with a cuff around the forearm. A computer calculates the velocity of the pulswave, augmentation index and central blood pressure simultaneously. Mobile Arteriographs are present at NUS, Clinical Research Center (KFC) and Östersund Hospital, Clinical Physiological Department. The latter will also be used at Sundsvall site. The PWV-device also measures the blood pressure. All subjects are requested to refrain from tobacco and drinking beverages containing caffeine 12 hours before examination, which will be performed in supine position, the dominant arm, in a silent room with air temperature 22–24°C. The measurements will be performed by the same and trained personel and at the same time of the day.

Size determination of hyaluronan in serum is performed by gas-phase electrophoretic mobility molecular analysis (GEMMA) (38). The GEMMA method is superior all other available methods for size determination of hyaluronan. It provides the same size separation as other methods but is considerable more sensitive, which allows for analysis of very small tissue samples, such as heart biopsies and skin biopsies. 10 to 20 mg tissue samples are being used which then after hyaluronan extraction need to be diluted 20 times before analysis with GEMMA. Furthermore, GEMMA is used for concentration estimation of hyaluronan in samples. A reliable molecular weight estimation of hyaluronan is achieved with less than 20 pico-gram of hyaluronan in solution diluted to 50 ng/mL or less.

- **Study Population**
- **Number and selection of Subjects**

Patients with RA (n=24) and SLE (n=24) will be recruited from the Rheumatology depart-ments in Umeå, Sundsvall, Sunderbyn and Östersund. 12 patients with RA and 12 patients with SLE will be randomized to each group. The randomization will be performed by study nurses, not otherwise involved in the conduction of the study, so that patients with RA receive even study numbers (Group 1 or Group 2) and patients with SLE (Group 1 and Group 2) receive uneven study numbers. A randomization list definining each study numbers group identity/treatment regim is prepared in advance by the study team.

- **Eligibility Criteria**
- **Inclusion Criteria**
- Male and female, 18-65 years that have given their written consent to participate in the study; Women of Childbearing Capacity (WOCBC) only if willing to comply with effective contraception methods during the course of trial . Acceptable methods are such as oral contraceptives, contraceptive patches, contraceptive implant, vaginal contraceptive, double-barrier methods (for example, condom and spermicide), intrauterine device (IUD), hormonal IUD.
- ACR criteria for RA and SLE (4)
- < 10mg Prednisolon daily doses
- Low-medium disease activity = DAS28 <4.6-RA, SLEDAI <6 SLE (Arthritis accepted)
- **Exclusion Criteria**

A subject will not be eligible for inclusion in this study if any of the following criteria applies:

- Antimalarial medication less than 5 ½-t i.e. 250 days before inclusion visit
- High disease activity, where cortison adjustment is predictable
- Pleuritis or Pericarditis – SLE
- Impaired visus and/or color vision
- Auditory nerve damage
- Cardiomyopathy
- Brach block / atrioventricular block
- Hypertension >160/95
- Diabetes
- Ongoing medical treatment with digoxin
- Re-introduction or recent (within 2 weeks) change in the dosing of NSAIDs, prednisolon, blood pressure lowering medication or antilipide treatment and within 6 weeks for disease-modifying agents
- Serious comorbidity, including other medications with known drug interactions with medication intended in the present study
- Short life expectancy due to other comorbidity
- Documented allergy or intolerance to the study drug.
- Severe psychiatric condition or other reason that jeopardize compliance with visit schedule
- In case of women of child bearing capacity (WOCBC), pregnancy assessed by anamnesis and pregnancy test
- **Other Eligibility Criteria Considerations**

To assess any potential impact on subject eligibility with regard to safety, the investigator must refer to the following documents for detailed information regarding warnings, precautions, contraindications, adverse events (AEs) and other significant data pertaining to study drug: Approved national summary of product characteristics (SPC).

- **Treatment within the study**

Hydroxychloroquine (Plaquenil®) 200 mg (Attachment 2. Summary of Product Characteristics) one tablet daily is initiated week 0 for group 1 and at week 5 for group 2and will be continued after the study is closed. All other medication continues during study, but if changes is performed in any medication, that might be a reason for discontinuation in the study (se also Section12.).

- **Withdrawal Criteria and Early Termination**

The subjects have the right to withdraw from the study at any time and for any reason without prejudice to his or her future medical care by the physician or hospital. The investigator also has the right to withdraw subjects from the study in the event of change in eligibility or other reasons.

Discontinuation criteria for individual subjects:

- Withdrawal of consent
- Changes in glucocorticoids, NSAID, ASA as well as medication against hyperlipidemia and hypertension
- Adverse event or serious adverse event related to treatment or investigational procedure that requires discontinuation in the study according to the investigator
- Pregnancy during the study
- **Study Assessments**
- **Demographic and Baseline Assessments**

Demographic information such as gender, age and ethnic origin will be collected in addition to baseline values.

Other baseline assessments are:

- Signed and dated ICF
- Inclusion/exclusion criteria
- Disease history/duration
- Current medication
- Disease activity by index (SLEDAI-2k or DAS 28)
- Vital signs
- WOCBC, pregnancy test
- Visus and color vision
- Blood lipids and-glucos
- See also Schedule of events, Attachment 1.
- **Assessments during the study**

Assessments include blood lipids, vascular stiffness, Calprotectin and Hyaluronan as described in 7.1 and 7.2 and 9.1.
In addition;

- Compliance to treatment
- Adverse events
- Compliance to drug prescription
- **Biobank**

Biobank is available at the Rheumatology Clinic, NUS, Umeå, Sweden (SoS443)

- **Study medication and concomitant medication/therapy**
- **Study medication per treatment group**

Hydroxychloroquine (Plaquenil®) 200 mg one tablet daily. See also risk-benefit for the individuals (4.2).

- **Administration follow-up of the medication:**

The responsible physicians for the patients will prescribe the study medication to the patients. In case of an adverse event, the study personnel will contact the sponsor. The sponsor will determine whether the event qualifies to immediate reporting to the regulatory authorities (SUSARs) or will be included to the annual development safety update report to Regulatory Authority (SAE). SAEs occurring after trial termination must be reported if considered related to the treatment during the trial.

- **Concomitant Medications**

The prohibited medication are considered at the time of inclusion (see section 10.2.2 “Exclusion Criteria”) and prevent the inclusion of patient not compliant to inclusion/exclusion criteria due to such ongoing medication.

During the study, all concomitant medications taken in both groups studied will be recorded in the patient record according to standard practice. In case a prohibited medication is noticed it results to patient withdrawal from the study (see sections 11. “Treatment within the Study” and 12. “Withdrawal Criteria and Early Termination”).

- **Adverse Event and Serious Adverse Event Collection, Recording and Reporting**

Known complications of RA and SLE will not be considered as a AE/SAE.

The investigator is responsible for detection and documentation of events meeting the criteria and definition of an adverse event (AE) or serious adverse event (SAE), as provided in this protocol. During the trial when there is a safety evaluation, the investigator or site staff will be responsible for reporting AEs and SAEs, as detailed in this section of the protocol. Clinically significant AEs considered by the investigator to be related to treatment will be followed until resolved or considered stable. It will be left to the investigator’s clinical judgment to determine whether an AE is related and of sufficient severity to require the subject’s removal from treatment or from the trial. A subject may also voluntarily withdraw from treatment due to what he or she perceives as an intolerable AE. If either of these situations arises, the subject should be strongly encouraged to undergo an end of trial assessment and be under medical supervision until symptoms cease or the condition becomes stable.

- **Definition of Adverse Events and Serious Adverse Events**
- **Definition of an Adverse Event**

An AE is defined as any untoward medical occurrence in a clinical trial subject administered a medicinal product and which does not necessarily have a causal relationship with this treatment. An AE can therefore be any unfavorable and unintended sign (including an abnormal laboratory finding), symptom, or disease (new or exacerbated) temporally associated with the use of a medicinal product, whether or not considered related to the product. For marketed medicinal products, this also includes failure to produce expected benefits (i.e. lack of efficacy), abuse or misuse.

- **Definition of a Serious Adverse Event**

A SAE is defined as any untoward medical occurrence or effect, that at any dose:

- Results in death
- Is life threatening
- Requires hospitalization or prolongation of existing hospitalization
- Results in persistent or significant disability or incapacity
- Is a congenital anomaly or a birth defect
- Is an other significant medical hazard

An AE necessitating hospitalization meets the regulatory definition for “serious” if the inpatient hospital admission includes a minimum of an overnight stay in a health care facility. Any AE that does not meet one of the definitions of serious (e.g. an AE requiring an emergency room visit, outpatient surgery or requires urgent investigation) may be considered by the investigator to meet the “other significant medical hazard” criterion for classification as a SAE. Examples include allergic bronchospasm, convulsions and blood dyscrasias.

- **Definition of a Suspected Unexpected Serious Adverse Reaction**

A SUSAR is both unexpected (not consistent with the applicable product safety reference information) and also meets the definition of a Serious Adverse Event/Reaction. The causal relationship with the treatment is defined as possible by the investigator.

For the purpose of this trial, the reference safety information defining what is to be considered as SUSAR is the Summary of Product Characteristics of Plaquenil (attachment 2).

- **Disease-Related Events and/or Disease-Related Outcomes Not Qualifying as AEs/SAEs**

Expected signs of disease activity such as joint stiffness and joint pain in RA and SLE or exantema in SLE that are well defined as disease related and do not qualify as AEs/SAEs.

- **Clinical Laboratory Abnormalities and Other Abnormal Assessments as AEs and SAEs**

Abnormal laboratory findings (e.g. clinical chemistry, hematology and urinalysis) or other abnormal assessments that are judged by the investigator as clinically significant will be recorded as AEs or SAEs if they meet the definition of an AE or SAE. Clinically significant abnormal laboratory findings or other abnormal assessments that are detected during the trial or are present at baseline and significantly worsen following the start of the trial will be reported as AEs or SAEs. However, clinically significant abnormal laboratory findings or other abnormal assessments that are associated with the disease being studied, unless judged by the investigator as more severe than expected for the subject’s condition, or that are present or detected at the start of the trial and do not worsen, will not be reported as AEs or SAEs.

The investigator will exercise his or her medical and scientific judgment in deciding whether an abnormal laboratory finding or other abnormal assessment is clinically significant.

- **Reporting of Adverse Events**

The investigator is responsible for ensuring that all AEs (as defined in Section 11.1) observed by the investigator or reported by subjects are properly captured in the subjects’ medical record. In addition, the investigator is responsible for ensuring that all AEs captured on the subjects’ medical records are reported on the CRF. If a subject is permanently withdrawn from the trial because of an SAE, this information must be included in the initial or follow-up SAE report form, as well as the End-of-Trial form. In case of withdrawal due to an AE, then the AE report form and as well as the End of Trial form will be completed. AEs either reported by the patient or observed by the investigator should be described in the following manner:

- The nature of the event will be described in precise, standard medical terminology (i.e. not necessarily the exact words used by the patient). If known, a specific diagnosis should be stated.
- The intensity of the AE will be described in terms of **mild, moderate or severe** according to the investigator’s clinical judgment. The intensity must be independent of the assessment of the seriousness of the AE.

**Mild:** Transient symptoms, no interference with the subject’s daily activities, acceptable.

**Moderate:** Marked symptoms, moderate interference with the subject’s daily activities but still acceptable.

**Severe:** Considerable interference with the subject’s daily activities, unacceptable.

The duration of the event will be described by the start date and end date.

**Related**: Related AEs or SAEs are classified as adverse reaction (AR) or serious adverse reaction (SAR).

**Probable**: Good reason and sufficient documentation to assume a causal relationship between prescribed medication and the AE. Probable related AEs or SAEs are classified as adverse reaction AR or SAR.

**Possible**: A causal relationship is likely and cannot be excluded. Possible related AEs or SAEs are classified as AR or SAR.

**Unlikely**: The event is most likely related to etiology other than any of the study drugs.

**Unknown**: Impossible to assess e.g. because of insufficient evidence, conflicting data or poor documentation.

**Not related**: No relationship to any of the study drugs.

The outcome of the event will be described in terms of:

**Recovered/resolved**

**Recovering/resolving**

**Not recovered/not resolved**

**Recovered/resolved with sequelae**

**Fatal**

**Unknown**

- **Time Period, and Frequency of Detecting AEs and SAEs/SUSARs**

From the time a patient consents to participate in the trial until he/she has completed the trial (without any follow up period), all AEs will be reported in the CRF. All SAEs will be reported promptly to *the sponsor*. SAEs will be reported by filling out and sending the SAE form within 24 hours to *the monitor* once the investigator determines that the event meets the protocol definition of an SAE. *The sponsor* will determine whether to whether the immediate notification to the regulatory authorities is required. SAEs occurring after trial termination must be reported if considered related to the treatment during the trial.

After the initial SAE report the investigator is required, proactively, to provide further information regarding the patient’s condition. All follow-up information must be forwarded to *the sponsor and monitor* as it becomes available. The investigator must follow-up all patients with SAEs until the event has subsided (or disappeared), the condition has stabilized, the event is otherwise explained or the patient is lost to follow-up.

For all deaths reported at any time, available autopsy reports and relevant medical reports should be forwarded to *the sponsor*.

Information about a suspected unexpected serious adverse reaction (SUSAR) which occurs during the course of the trial and is fatal or life-threatening will be reported *by the sponsor* as soon as possible to the regulatory authority (directly, and via them to EudraVigilance Clinical Trials Module) and the relevant ethics committee.

This will be done no later than 7 days after *the investigator* first became aware of the reaction. Any additional relevant information should be sent within 8 days of the initial report. A SUSAR, which is not fatal or life threatening will be reported by *the sponsor* as soon as possible, and no later than 15 days after becoming aware of the reaction to the competent authorities (directly, and via them to EudraVigilance Clinical Trials Module) and the relevant ethics committee.

- **Pregnancies and Breast Feeding**

Subjects who become pregnant during the trial must be withdrawn. The subject will be asked to attend follow-up visits and the outcome of the pregnancy will be followed including reporting on the state of the child 8 to 12 weeks after delivery. Patients receiving treatment shall not breast feed their children.

- **Data Analysis and Statistical Considerations**
- **Sample Size Considerations**

Twenty patients in each group are needed for a 80%-ig power of lowering blood-pressure 10 mmHg. Lowering LDL or TC 0.65 mmol/L with SD 0.7 gives a power of 0.816 when 20 pati-ents in each group are included.

- **Data Sets and Populations**

All subjects enrolled will constitute the analysis population.

- **Interim Analysis**

No interim analysis will be performed

- **Key Elements of Analysis Plan**

Non-parametric tests will be used. Mann-Whitney´s test; when comparison between groups. Statistical testing of multiple assessments over time; Kruskal-Wallis or Friedmans test and Wilcoxons test.

- **Study Administration**
- **Procedure for obtaining informed consent**

Eligible individuals in the study will be contacted by mail containing information about the study, informed consent and return envelope.

- **Definition of Source Data**

All completed assessment scales will be collected in patient specific binders and regarded as source data. All patient related activities of medical relevance will also be documented in the patient hospital record

- **Quality Control**

The trial will be monitored consistent with the demands of the trial and site activity to verify that the:

- Safety and rights of subjects are being protected
- Data are authentic, accurate, and complete
- Trial is conducted in accordance with the currently approved protocol and any other trial agreements, GCP and all applicable regulatory requirements. The investigator and the head of the medical institution (where applicable) agrees to allow *the monitor* direct access to all relevant documents.

An external monitor for the study, not involved in any aspect of the treatment of the individuals participating in the study, will be appointed. The monitor will be appropriately trained, and has the scientific and/or clinical knowledge needed to monitor the trial adequately.

The monitoring will take place in accordance to GCP before, during and after the trial. If any misconduct or neglect would be notified the sponsor and investigator will be notified for to corrective and preventive actions.

- **Quality Assurance**

It is the investigator´s responsibility:

- That he CRFs are reviewed for completeness and accuracy and the appropriate CRF pages also are signed and dated.
- To ensure compliance with GCP and all applicable regulatory requirements, regulatory agencies may conduct a regulatory inspection of this trial. Such audits/inspections can occur at any time during or after completion of the trial. If an audit or inspection occurs, the investigator and institution agree to allow the audit/inspection.
- **Study Closure**

The study will be formally closed when after 12 weeks from study start.

The investigator is fully responsible to consciously monitor safety and make any decisions regarding premature closure of the study because of safety issues

- **Records Retention**

The individuals participating in the study will be coded as serial numbers which means that when summarizing the data, it will not be possible to see which results belong to which individual without access to the coded list with serial numbers. The results will be presented at group level without tracing to the respective individual. The case record files will be stored in locked facilities at the Rheumatology department, Östersund Hospital, Sweden. The randomization will be conducted by study nurses at Östersund Hospital, Rheumatology Unit, not otherwise involved in conduction of this study.

Following closure of the study, the investigator or the head of the medical institution (where applicable) must maintain all site study records, except for those required by local regulations to be maintained by someone else, in a safe and secure location. The records must be maintained to allow easy and timely retrieval, when needed and, whenever feasible, to allow any subsequent review of data in conjunction with assessment of the facility, supporting systems and staff.

The archiving time for the study documents is at least 10 years after the trial has been completed and the study report compiled.

- **Case Report Forms**

A simplified CRF for each participating subject will be manufactured by the investigator and collected in patient specific binders.

- **Rules for completing CRFs**

Use a blackpoint or bluepoint pen when completing the CRFs. Ensure that all relevant questions are answered and that no empty answer fields exist after each visit.

If a test/assessment has not been done and will not be available a straight line should be drawn across the question module and write ‘ND’ (Not Done). If the question is irrelevant indicate this by writing ‘NA’ (Not Applicable). If a value or variable is not known it will be indicated ‘NK’ (Not Known)

The study personnel must ensure that all information entered into the CRF is consistent with the source data.

- **Corrections to CRFs**

Corrections are done by drawing a straight line through the incorrect data. Write the correct data next to the one that had been crossed out. Confirm the correction by writing your initials and the date the correction was done.

If a correction need to be done after the original CRF had been removed and sent to data management, the correction is done by using a Query Resolution Form. This form can be sent by data management when any entered information need to be clarified or when information is missing.

- **CRF flow**

The study personnel are responsible to complete the CRF after each subject visit.

- **Ethical and legal aspects**
- **Study ethics**

This study will be conducted in compliance with the ethical principles that have their origins in the Declaration of Helsinki, Good Clinical Practice, applicable regulatory requirements and this protocol.

- **Study approval, amendments and reporting to authorities**

The study must not start before written approvals by the Ethical Committee and Competent Authority has been obtained.

Changes to the approved final study protocol will be documented in written and numbered Protocol Amendments. The amendments should be signed and dated by the same parties who signed the final protocol, as applicable. All essential changed has to be approved by the Ethical Committee and Competent Authority.

An annual development safety update report (DSUR) will be sent to regulatory authority. Declaration of End of Trial Notification will send by sponsor to regulatory authority within 90 days after end of study.

- **Data Management**

Statistical analyses (see 6.1) will be performed by the investigators.

- **Finance and Insurance**

The study is not sponsored by any pharmaceutical company but financed by research grants.

Every subject participating in the study is covered by his or her national insurance as a patient (Patienskadeförsäkring, Läkemedelsförsäkring).

- **Reference**
- Wållberg-Jonsson S et al. Cardiovascular mortality and morbidity in patients with seropositive rheumatoid arthritis in Northern Sweden. J Rheumatol. 1997 Mar;24(3):445-51.
- Urowitz MB et al. The bimodal mortality in SLE. Am J Med 1976;60:221-5.
- Bengtsson C et al. Cardiovascular event in systemic lupus erythematosus in northern Sweden: incidence and predictors in a 7-year follow-up study. Lupus. 2012 Apr;21(4):452-9.
- Wållberg-Jonsson S et al. Increased arterial stiffness and indication of endothelial dysfunction in longstanding rheumatoid arthritis. Scand J Rheumatol. 2008 Jan-Feb;37(1):1-5.
- Södergren A et al. Atherosclerosis in early Rheumatoid Arthritis: Very early Endothelial Activation and Rapid Progress of Intima Media Thickness Arthritis Res Ther. 2010;12(4):R158.
- Bjarnegård N et al. Increased aortic pulse wave velocity in middle aged women with systemic lupus erythematosus Lupus. 2006;15(10):644-50.
- Wållberg-Jonsson S et al. Extent of inflammation predicts cardiovascular disease and overall mortality in seropositive rheumatoid arthritis. J Rheumatol. 1999 Dec;26(12):2562-71.
- Dessein PH et al. Biomarkers of endothelial dysfunction, cardiovascular risk factors and atherosclerosis in rheumatoid arthritis. Arthritis Res Ther. 2005;7(3):R634-43.
- Urowitz MB et al. Atherosclerotic vascular events in a single large lupus cohort: prevalence and risk factors. J Rheumatol 2007;34:70–5.
- Gustafsson J et al et al. Predictors of the first cardiovascular event in patients with systemic lupus erythematosus – a prospective cohort study. Arthritis Res Ther 2009;11(6):R186.
- Moreland LW et al. A randomized comparative effectiveness study of oral triple therapy versus etanercept plus methotrexate in early aggressive rheumatoid arthritis: the treatment of Early Aggressive Rheumatoid Arthritis Trial. Arthritis Rheum. 2012 Sep;64(9):2824-35.
- Meinão I et al. Controlled trial with chloroquine diphosphate in systemic lupus erythematosus. Lupus 1996;5:237–41.
- Tsakonas E et al. A long-term study of hydroxychloroquine withdrawal on exacerbations in systemic lupus erythematosus. The Canadian Hydroxychloroquine study group. Lupus 1998;7:80–5.
- Nikpour M et al. Variability over time and correlates of cholesterol and blood pressure in systemic lupus erythematosus: a longitudinal cohort study Arthritis research & therapy 2010;12:R125.
- Selzer F et al. Vascular stiffness in women with systemic lupus erythematosus. Hypertension 2001;37:1075–82.
- Jung H et al. The protective effect of antimalarial drugs on thrombovascular events in Systemic Lupus Erythematosus. Arthritis & Rheumatism 2010;62:863-8.
- Munro R et al. Effect of disease modifying agents on the lipid profiles of patients with rheumatoid arthritis. Ann Rheum Dis. 1997 Jun;56(6):374-7.
- Morris SJ et al. Hydroxychloroquine use associated with improvement in lipid profiles in rheumatoid arthritis patients. Arthritis Care Res (Hoboken). 2011 Apr;63(4):530-4.
- Cairoli E et al. Hydroxychloroquine reduces low-density lipoprotein cholesterol levels in systemic lupus erythematosus: a longitudinal evaluation of the lipid-lowering effect. Lupus 2012;11:1178-82.
- Laurent, T.C. & Fraser, J.R. Hyaluronan. FASEB J 6, 2397-2404 (1992).
- Lee, J.Y. & Spicer, A.P. Hyaluronan: a multifunctional, megaDalton, stealth molecule. Curr Opin Cell Biol 12, 581-586 (2000).
- Toole, B.P. Hyaluronan is not just a goo! J Clin Invest 106, 335-336 (2000).
- Delmage, J.M et al. The selective suppression of immunogenicity by hyaluronic acid. Ann Clin Lab Sci 16, 303-310 (1986).
- McBride, W.H. & Bard, J.B. Hyaluronidase-sensitive halos around adherent cells. Their role in blocking lymphocyte-mediated cytolysis. J Exp Med 149, 507-515 (1979).
- Asari, A. Novel Functions of Hyaluronan Oligosaccharides. Science of Hyaluronan Today. [http://www.glycoforum.Ann.gr.jp/science/hyaluronan/HA12a/HA12aE.html](http://www.glycoforum.ann.gr.jp/science/hyaluronan/HA12a/HA12aE.html) (2005).
- Laurent, TC, Laurent UB, Fraser JR. Function of hyaluronan. 1995. Ann Rheum Dis, 54:429-32.
- Marshak-Rothstein A. Toll-like receptors in systemic autoimmune disease. Nat Rev Immunol 2006;6: 823-35.
- Crispin J. Tsokos C. Novel molecular targets in the treatment of systemic lupus erythematosus. Autoimmun Rev. 2008 Jan;7(3):256-61.
- Okamoto T. NF-kappaB and Rheumatic diseases. Endocr Metab Immune Disord Drug Targets 2006 Dec;6(4):359-72.
- Wallace DJ et al. New insights into mechanisms of therapeutic effects of antimalarial agents in SLE. Nat Rev Rheumatol. 8,522-33 (2012).
- Lindqvist U, Pihl-Lundin I, Engström-Laurent A. Dermal distibution of Hyaluronan in psoriatic arthritis; coexistense of CD44, MMP3 and MMP9. Acta-Dermatol-venerologica 2012 Jul;92(4):372-7.
- Kubo M et al. Increased serum concentration of hyaluronate in dermatomyositis patients. Arch Dermatol Res 290:579-81, 1998.
- Yung S, Chan TM. The role of Hyaluronan and CD44 in the pathogenesis of Lupus Nephritis. Autoimmune Diseases. Autoimmune Diseases. 2012;2012:207190. doi: 10.1155/2012/207190. Epub 2012 Aug 1.
- Gladman DD, Iban˜ ez D, Urowitz MB. Systemic lupus erythematosus disease activity index 2000. J Rheumatol 2002;29:28891.
- Prevoo MLL et al. Modiﬁed disease activity scores that include twentyeight-joint counts: development and validation in a prospective longitudinal study of patients with rheumatoid arthritis. Arthritis Rheum 1995;38:44 –8.
- C Lood et al. Protein synthesis of the pro-inflammatory S100A8/A9 complex in plasmacytoid dendritic cells and cell surface S100A8/A9 on leukocyte subpopulations in systemic lupus erythematosus. Arthritis Research & Therapy 2011;13;R60.
- Horváth I G et al. Invasive validation of new oscillometric device (Arteriograph) for measuring augmentation index, central blood pressure and aortic pulse wave velocity. Journal of hypertension 2010;28(10):2068-75.
- Malm L, Hellman U, Larsson G. Size determination of hyaluronan using a gas-phase electrophoretic mobility molecular analysis. Glycobiology. 2012 Jan;22(1):7-11.

Hage MP, Al-Badri MR, Azar ST. A favorable effect of hydroxychloroquine on glucose and lipid metabolism beyond its anti-inflammatory role.Ther Adv Endocrinol Metab. 2014 Aug;5(4):77-85.

Wållberg-Jonsson S, Caidahl K, Klintland N, Nyberg G, Rantapää-Dahlqvist S. Increased arterial stiffness and indication of endothelial dysfunction in longstanding rheumatoid arthritis. Scand J Rheumatol. 2008 Jan-Feb;37(1):1-5

López-Pedrera C1, Aguirre MÁ, Barbarroja N, Cuadrado MJ

Accelerated atherosclerosis in systemic lupus erythematosus: role of proinflammatory cytokines and therapeutic approaches. J Biomed Biotechnol. 2010; Epub 2010 Sep 26.

ATTACHMENT 1. SCHEDULE OF EVENTS

| **Procedures** | **Screening**  **Week -2;** for eligible patients thereafter **Randomization** | **Week 0** | **Week 4** | **Week 8** | **Week 12 #** |
| --- | --- | --- | --- | --- | --- |
| Informed consent | X |  |  |  |  |
| Inclusion/Exclusion  Criteria | X |  |  |  |  |
| SLEDAI-2k (SLE) | X |  |  |  |  |
| DAS 28 (RA) | X |  |  |  |  |
| Medical history and present medication | X | X |  |  |  |
| Vital signs (pulse and blood pressure) | X |  |  |  |  |
| Pregnancy test* | X |  |  |  |  |
| Visus Color vision | X |  |  |  |  |
| Blood samples: |  |  |  |  |  |
| Chol | X | X | X | X | X |
| TG | X | X | X | X | X |
| HDL | X | X | X | X | X |
| LDL | X | X | X | X | X |
| ApoA1 |  | X | X | X | X |
| ApoB |  | X | X | X | X |
| Lp(a) |  | X | X | X | X |
| HbA1c | X | X | X | X | X |
| B-glc | X | X | X | X | X |
| CRP |  | X | X | X | X |
| Serum for storage  -20◦C. |  | X | X | X | X |
| PWV including  Pulse and blood  pressure |  | X | X | X | X |
| Adverse events |  |  | X | X | X |
| Compliance to drug prescription |  |  | X | X | X |

* For WOCBC

# Control group
